# White Matter Injury and Recovery after Hypertensive Intracerebral Hemorrhage

**DOI:** 10.1155/2017/6138424

**Published:** 2017-06-07

**Authors:** Shilun Zuo, Pengyu Pan, Qiang Li, Yujie Chen, Hua Feng

**Affiliations:** Department of Neurosurgery, Southwest Hospital, Third Military Medical University, Chongqing, China

## Abstract

Hypertensive intracerebral hemorrhage (ICH) could very probably trigger white matter injury in patients. Through the continuous study of white matter injury after hypertensive ICH, we achieve a more profound understanding of the pathophysiological mechanism of its occurrence and development. At the same time, we found a series of drugs and treatment methods for the white matter repair. In the current reality, the research paradigm of white matter injury after hypertensive ICH is relatively obsolete or incomplete, and there are still lots of deficiencies in the research. In the face of the profound changes of stroke research perspective, we believe that the combination of the lenticulostriate artery, nerve nuclei of the hypothalamus-thalamus-basal ganglia, and the white matter fibers located within the capsula interna will be beneficial to the research of white matter injury and repair. This paper has classified and analyzed the study of white matter injury and repair after hypertensive ICH and also rethought the shortcomings of the current research. We hope that it could help researchers further explore and study white matter injury and repair after hypertensive ICH.

## 1. Introduction 

Hypertension is one of the three leading risk factors for global disease burden [1], which is known to be a basic risk factor for stroke. In 2010, approximately 16.9 million incident strokes occurred, which added up to a pool of 33 million stroke survivors worldwide [2, 3]. Hemorrhagic stroke accounted for about 31.52% of all strokes, and the most common origin is hypertension (30–60%) [2]. The most frequent occurrence location of hypertensive ICH is around the basal ganglia and thalamus, which could easily lead to death or disability.

White matter fibers, especially located within the capsula interna, are one of the most vulnerable tissues in hypertensive ICH. Quantities of pathogenic factors, which are generated by hypertensive ICH, could impact the structure and function of white matter fibers. A study found that white matter injury might reflect the vulnerability of individual brains to pathologic insults and suggested that it should be considered when assessing immediate, early, and long-term outcomes after ICH [4]. At present, white matter injury and repair after hypertensive ICH have drawn more and more attention from researchers. However, the current study of white matter injury and repair after hypertensive ICH is still scanty and scattered. Besides, animal research showed that scores of therapeutic agents and methods were effective in the treatment of white matter injury after hypertensive ICH, while these drugs and treatment methods are rarely used in clinical practice. For one thing, it is the deficiency of animal model; for another, we need to rethink our research strategy on white matter injury and repair after hypertensive ICH.

There is no animal model able to completely simulate the natural process of human white matter injury after hypertensive ICH at present. Each animal model has its drawbacks, but each one could be used to meditate on certain pathophysiological aspects of the white matter injury after hypertensive ICH. On the other hand, as it is known, the white matter might be simultaneously or sequentially damaged by pathogenic factors which are evoked by hypertensive ICH. Owing to that, the work for a single pathogenic factor does not have an edge on the effective treatment of white matter injury and promoting its recovery. And these pathogenic factors could also damage the other brain tissues around the damaged white matter, as blood vessels and nerve nuclei [5, 6]. In terms of functioning, brain structures influence and interact with each other. They are operating as a whole. Unfortunately, we inspected them separately. In an effort to overcome these shortcomings, we need a new research paradigm which should consist of a bleeding artery, the damaged white matter and its peripheral nerve nuclei, and so forth.

In this review, we initially depict the white matter injury after hypertensive ICH in detail, followed by a thorough elaboration of the current strategies for control of the white matter injury. Thereafter, we comment on past and current relevant studies and finally propose the lenticulostriate artery-neural complex (LNC) as a new research paradigm.

## 2. White Matter Injury after Hypertensive ICH

### 2.1. The General Epidemiology of White Matter Injury after Hypertensive ICH

White matter injury, which is caused by hypertensive ICH, occurs rather rapidly. For example, hypertensive ICH could immediately cause hemorrhagic hypotension, which reduces the systemic arterial blood pressure by 35–45% [7]. During this period, the lowest cerebral blood flows were in the white matter [7], while blood flows to all brain regions would increase by 31–42% over steady-state values after 5 minutes of hemorrhagic hypotension [7]. Dramatic changes of cerebral circulation within such a short period of time could damage the white matter and then affect the level of consciousness and other cerebral functions [8]. Thus, aggressive and early medical intervention is necessary to reduce white matter injury after hypertensive ICH.

The main location of white matter injury and that of hypertensive ICH are closely related. Different cerebral structures (such as blood vessels, white matter, and nerve nuclei) exhibited differential insensitivity to the effects of hypertension. Therefore, the occurrence of hypertensive ICH has obvious anatomic sites preference. A study in China found that hypertension was seen in 79.1% of basal ganglia and 68.2% of thalamic ICH patients, but in only a minority of cerebellar (22.2%) and lobar (20.2%) ICH cases [9]. Moreover, the majority of single hemorrhages were found in deep (subcortical) sites, including the basal ganglia (34.2%), thalamus (8.3%), cerebellum (6.8%), ventricles (1.5%), and brainstem (1.1%) [9]. Hence, around the basal ganglia and the thalamus are the main sites of white matter injury after hypertensive ICH. Research on white matter injury after hypertensive ICH should focus on these sites in the future.

The damaged degree of white matter is determined by the spatial and temporal relationship with the hypertensive ICH. A study pointed out that, one day after ICH, massive dMBP^+^ (degraded myelin basic protein) white matter tracts were seen in the core and at the edge of the hematoma, whose morphology was relatively normal; by 3 days, dMBP staining was lighter and the white matter tracts were more fragmented and larger; at a later time after ICH (28 days), dMBP was not detected in the ipsilateral striatum in laboratory animals, even inside or at the edge of the lesion [10]. But the area with dMBP increased between 6 hours and 1 day in the ICH model but did not exhibit further changes by 3 days [10]. Interestingly, the authors did not detect dMBP in the parenchyma outside the hematoma at any of the time points [10]. But other evidence suggested that the existence of early moderate ischemia could cause white matter lesion, in the parenchyma outside the hematoma. For instance, multimodal monitoring demonstrated hematoma volume-dependent changes of tissues oxygenation, blood flow, and ischemic microdialysis markers in the gray-white matter junction for 12 hours of monitoring [11]. This reminds us of the issue of whether a slight white matter lesion existed in the parenchyma outside the hematoma, which requires further research in the future work.

### 2.2. Pathogenic Factors of White Matter Injury after Hypertensive ICH

**Figure 1:**
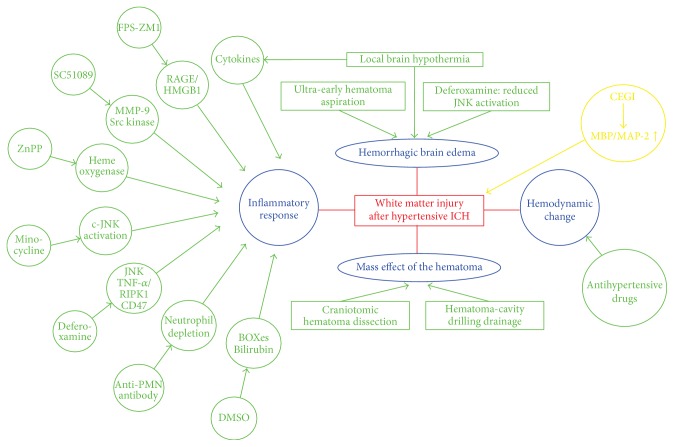
Schematics for the pathogenic factors of white matter injury after ICH.

Besides the direct cell death (including but not limited to apoptosis, necrosis, autophagy, and recently necroptosis) in oligodendrocytes, myelin, or axon, a good many pathogenic factors (Figure 1) could be involved in white matter injury after hypertensive ICH, such as the hemorrhagic brain edema, the mass effect of the hematoma, the hemodynamic change of the cerebral circulation, and the inflammatory response induced by the blood components and metabolites. The white matter is simultaneously or sequentially damaged by these pathogenic factors after hypertensive ICH, while other damaged brain tissues around the white matter could conversely generate cell death of white matter components.

The hemorrhagic brain edema after hypertensive ICH could quickly reduce the metabolic rate and then cause injury of the white matter. Animal experiments showed that, compared with the control group, glycogen and glucose concentrations, respectively, increased twofold to fivefold during a period of 8 hours after infusion; phosphocreatine levels increased severalfold by 5 hours; and lactate was obviously increased (approximately 20 mMol/g) at 1, 3, 5, or 8 hours after infusion in markedly edematous white matter [12]. In addition, the hemorrhagic brain edema after hypertensive ICH occurs promptly and lasts longer. Previous studies in pig models indicated that edematous white matter areas were present directly around the hematoma at 1 hour after ICH [13]. This region had a greater than 10% increase in water content (>85%) compared with the contralateral white matter (73%), and this increased water content persisted through 8 hours [13]. And a clinical study found that the patient's edema volume at the second week went up in comparison with the first week after hypertensive ICH; and the edema volume in the fourth week only returned to the same level as in the first week [14]. Sudden and prolonged brain edema after hypertensive ICH are mainly driven by blood vessel disruption and serum proteins and plasma proteins (such as albumin and IgG) accumulation in the white matter around the hematoma [13, 15–17]. Interestingly enough, hypertensive ICH could elevate the content of cerebral interstitial serum proteins through cerebral vessels about 3-fold compared with normotensive ICH in laboratory animals [18]. And later animal experiments found that serum proteins derived from blood clot formation in normotensive ICH [16]. This is an indication that white matter injury, which is caused by edema after hypertensive ICH, has its specialties and that distinctions in studies should be made in the future.

Another major etiological factor after hypertensive ICH is the mass effect of the hematoma, which could reduce CBF and then cause ischemic damage of the white matter. The mechanical microballoon model is applied frequently to simulate the effect. Previous research revealed that experimental animals exhibited significant ischemic damage and reduced CBF persisted for 4 hours after transient inflation of a microballoon in the caudate nucleus [19]. Clinical research also showed us that subcortical white matter might be damaged by the mass effect when the volume of the hematoma exceeds around 25 mL after hypertensive ICH [20]. And our recent findings suggested that the mass effect of the hematoma could cause direct pathologic damage to the white matter by the mechanical microballoon model in rats [21]. Although surgical efficacy of removal of intracerebral hematoma has always been controversial [22, 23], it is still valuable for us to research the relationship between the mass effect of hematoma and the white matter injury.

The hemodynamic change of the cerebral circulation is also one of the important causes for white matter injury after hypertensive ICH. Hemodynamic parameters experienced a dramatic shift in a short time after hypertensive ICH. As is stated above, hypertensive ICH could immediately cause hemorrhagic hypotension, which reduced the systemic arterial blood pressure by 35–45% in experimental animals [7]. Yet, a large number of clinical trials discovered that the mean systolic blood pressure within 3 hours of hypertensive ICH was substantially higher than premorbid levels (mean increase of 40.7 mmHg, *p* < 0.0001) [24]. These changes will inevitably affect the white matter blood flow. Animal experiments have shown that the lowest cerebral blood flows were in the white matter during the hemorrhagic hypotension period, but the blood flows of the white matter increased by 31–42% over steady-state values after 5 minutes [7], which is a serious injury to the white matter. Therefore, sustaining steady hemodynamics of cerebral circulation might be contributed to alleviate the white matter injury after hypertensive ICH.

After hypertensive ICH, the inflammatory response, which is induced by the blood components and metabolites, was severely destructive to the white matter. The components of plasma (such as thrombin, complement, glutamate, and carbonic anhydrase 1) and released substances from the hematoma (such as hemoglobin and iron) could cause inflammation and damage through promoting the activation of resident microglia and the production of inflammatory mediators by influx of leukocytes into the brain [25, 26]. For instance, animal research found that plasma protein led to rapid white matter injury through inducing a cascade of acute inflammatory events including oxidative stress, proinflammatory cytokine gene expression, and DNA damage within 24 h after ICH [27]. In addition, blood breakdown products could also cause white matter injury in a delayed manner, for a study found that bilirubin and bilirubin oxidation products could affect the structural integrity and function of white matter tracts of the corpus callosum at 7 days after ICH [28]. Accordingly, for the white matter injury after hypertensive ICH, we should pay attention to the delayed inflammatory response as well as acute inflammatory response in the future.

To sum up, the white matter injury after hypertensive ICH was caused by a variety of pathogenic factors. It is very difficult for single therapeutic strategies to prevent and treat white matter injury after hypertensive ICH. So, multiple therapeutic interventions and multipotential drugs should be taken seriously in the future work.

### 2.3. Pathological Changes of Cerebral White Matter after Hypertensive ICH

The pathological changes of white matter after hypertensive ICH were blatantly obvious. Recent animal experiments showed that the white matter was progressively lost in the perihematoma from day 1 to day 28 after ICH [29]. Myelin sheaths played an important role in maintaining the morphology and function of the white matter. Hypertensive ICH could lead to demyelination and downregulation of MBP expression in the white matter. Morphologic changes of myelin sheaths included swelling and damage at first, followed by demyelination and lastly oligodendrocyte apoptosis after ICH [30]. Accompanying this, a large number of pathological molecules were highly expressed or overactivated, such as TNF-*α* [31], RIPK1 [32], receptor for advanced glycation end-products (RAGE), HMGB1 [33], CD47 [34], SC1 [35], *β*-APP [36], and JNK signaling pathway [37]. These pathological molecules had aggravated the damage of myelin sheaths and then affected the morphology and function of the white matter. Meanwhile, it should be noted that the self-repairing function of brain tissue played an active role, as the densities of immature oligodendrocyte precursor cells (OPCs) and mature oligodendrocytes in the perihematoma increased dramatically over the first week after ICH in rats [38]. Therefore, it may be a better strategy to improve the self-repairing function of brain tissue in the treatment of white matter injury after hypertensive ICH.

### 2.4. Consequence of White Matter Injury on Patients after Hypertensive ICH

White matter injury will seriously affect the prognosis of hypertensive ICH patients. A study found that severe white matter injury is a prognostic factor for poor activities of daily living at discharge in elderly patients with stroke [39]; and severe leukoaraiosis is also independently associated with the long-term mortality in survivors after ICH [40]. The clinical manifestation of ICH patients is strongly connected with the location and the degree of white matter injury. As already mentioned above, around the basal ganglia and thalamus are the main sites of white matter injury after hypertensive ICH. White matter injury in this region could bring about persistent aphasia [20], buccofacial apraxia [41], ideational apraxia [42], dysgraphia [43], and so forth. But not all white matter injuries could contribute to these clinical features, just as the volume of hematoma exceeding around 25 mL is the precondition for the occurrence of white matter damage [20]. To conclude, although we found that white matter injury is closely related to some clinical features, there are relatively few studies in this field and the pathogenic mechanism is unclear. It is crucial to further explore the relationship between white matter injury and clinical manifestations.

### 2.5. Detection Methods for the White Matter Injury after Hypertensive ICH

The accurate detection of white matter injury helps in the prevention and treatment of hypertensive ICH (Table 1). At present, the most extensive detection technique used for white matter injury is magnetic resonance imaging (MRI). For detecting white matter injury, diffusion tensor imaging (DTI) and T1- and T2-weighted spin-echo sequences (T1WI and T2WI) could all be used as certain types of MRI [44–46]. DTI tractography was the most suitable for assessing longitudinal changes in white matter fibers' integrity and mechanical displacement [44, 47]. Pathology-affected white matter fibers in patients with ICH could be selectively visualized by using structural neuroimaging and DTI volumes [44]. Combined usage of T1WI and T2WI in patients with hypertensive ICH could discover white matter injury, such as widespread white matter edema [48], hemorrhagic lacunes [45], and white matter hyperintensities [49]. However, T1WI and T2WI could not intuitively understand the damage of white matter fibers compared with DTI. As a result, if accurate study of the white matter injury with MRI is required, DTI should be the top priority.

Computed tomographic (CT) scan is another technique for detecting white matter injury after hypertensive ICH. Being able to quickly identify and differentiate hemorrhagic stroke from ischemic stroke is of extremely important significance for the clinic. The main manifestation of white matter damage on CT is reduced white matter density (leukoaraiosis) [50, 51]. Significant leukoaraiosis has been found in about 38% of cerebral apoplexy patients [50]. In order to distinguish the degree of white matter damage more accurately, leukoaraiosis was scored on the baseline of CT scan as described by van Swieten et al., with an overall score from 0 to 4 [40, 52]. Although efforts had been made, CT is still unable to catch up with MRI in the detection of white matter injury. Therefore, the relevant research reports are few.

Pathological examination is the principal method for diagnosis. The pathological changes of white matter after hypertensive ICH could be quickly and accurately found by immunohistochemical staining, western blotting, southern blotting, PCR, and so forth [29, 30, 33]. The pathological changes of white matter after hypertensive ICH were involved not only in morphology, but also in function. Only pathological examination could perfectly solve these problems. Besides, pathological examination could also be used to assess the self-repairing function of the white matter after hypertensive ICH; namely, the densities of immature OPCs and mature oligodendrocytes in the perihematoma were determined by pathological examination [38]. Even though pathological examination has many advantages, as an invasive examination, it could not possibly be widely carried out in patients with hypertensive ICH.

In addition, there are indirect ways to detect white matter injury after hypertensive ICH; for example, white matter blood flow changes after hypertensive ICH could be detected using laser Doppler and the radioactive microsphere technique [7, 53]. Moreover, poststroke activities of daily living (ADL), Informant Questionnaire on Cognitive Decline in the Elderly (IQCODE), and the Modified Telephone Interview for Cognitive Status test could all be used to indirectly determine the degree of white matter injury of hypertensive ICH patients [39, 54, 55].

In summary, none of the methods above are flawless. It is of vital importance to find noninvasive and more accurate detection methods for further study of white matter injury after hypertensive ICH.

## 3. Current Strategies for White Matter Injury after Hypertensive ICH

### 3.1. Therapeutic Strategies for the Pathogenic Factors

As stated above, of the various pathogenic factors that resulted in white matter injury after hypertensive ICH, the mass effect of hematoma was the most vital one. Surgical removal of the hematoma could relieve oppression of the surrounding brain tissue, lower intracranial pressure, relieve and prevent cerebral hernia, improve cerebral blood flow, reduce sequela, and so forth. Hence, this is a good way. There are two main surgical methods for the removal of hematoma: one is the craniotomic hematoma dissection and the other is the hematoma-cavity drilling drainage [56]. Yet, there is no study on whether the surgical removal of the hematoma could reduce the damage of white matter at present. On the other hand, the hemorrhagic brain edema is another pathogenic factor causing white matter injury after hypertensive ICH. Blood clot formation could cause the occurrence of brain edema after ICH [16]. It was reported that ultra-early hematoma aspiration after fibrinolysis with t-PA in a porcine model of ICH notably reduced perihematomal edema and protected the blood-brain barrier [57]. This suggests that surgical removal of the hematoma may help reduce white matter injury through different mechanisms, which is worth of intensive study in the future.

Using drugs to regulate the expression and the related pathogenesis of pathogenic molecules is another strategy to reduce the damage of white matter after hypertensive ICH. Some drugs had been shown to reduce the damage of white matter in animal models of ICH, just as FPS-ZM1 (RAGE-specific antagonist) could relieve the damage of white matter via antagonism of ligand/receptor interaction [33]; SC51089 (EP1R antagonist) could decrease Src kinase phosphorylation and MMP-9 activity and then relieve the damage of the white matter [58]. Zinc protoporphyrin (ZnPP), as a heme oxygenase inhibitor, could also reduce white matter injury [29]; and minocycline was able to suppress Fe-induced white matter injury and c-JNK activation after hypertensive ICH in rats [36]. As a crucial component of the white matter, axon damage after ICH is relatively common. A study found that dimethyl sulfoxide or its structurally related derivatives, which could effectively attenuate the toxic effect of bilirubin and its oxidation products, might have a potential therapeutic value in antagonizing axonal damage after ICH [28].

Deferoxamine is a medicine which captures our attention. Previous animal research found that deferoxamine could remarkably reduce white matter injury after ICH via multiple mechanisms, including inhibiting white matter edema [37], reducing ICH-induced JNK activation [37], lowering TNF-*α* and RIPK1 levels [32], and upregulating the expression of CD47 [34]. In view of the good performance of deferoxamine in the animal experiment, a clinical trial was carried out and went on well. Currently, third-phase clinical trials of deferoxamine are underway. Hopefully, it would be widely used in clinical treatment in the foreseeable future.

In addition, two other interesting therapeutic methods are noticeable for white matter injury. One is the delayed profound local brain hypothermia. This study found that the delayed profound local brain hypothermia was able to protect white matter tracts through strikingly reduced inflammatory cytokines production and vasogenic edema development in a porcine model of ICH [59]. The other is neutrophil depletion. Circulating blood neutrophils were depleted with an anti-PMN antibody before inducing ICH in rats. After experiments, detailed spatial analysis showed that neutrophils depletion reduced infiltration of activated microglia/macrophages in the perihematoma white matter tracts and decreased myelin fragmentation and axon damage [17, 60].

### 3.2. Therapeutic Strategies for White Matter Restoration after Hypertensive ICH

Due to less attention on the restoration of white matter injury after hypertensive ICH in the past, there is not much evidence for white matter restoration after hypertensive ICH. Inspired by a few recent studies, endogenous or exogenous stem cell therapy [61, 62], as well as new drugs towards the pathogenic factors summarized in Section 2.2, may be the promising strategy to promote the repair of damaged white matter injury after hypertensive ICH. Joseph et al. found that endogenous oligodendrocyte precursors could proliferate and differentiate in the perihematoma region and had the potential to remyelinate axon tracts after ICH [38]. And another animal experiment also found that cattle encephalon glycoside and ignotin (CEGI) treatment could effectively upregulate MBP/MAP-2 expression, ameliorate white matter fibers damage, and alleviate the neurobehavioral dysfunction after ICH [63]. Minocycline and deferoxamine were also demonstrated to be protective towards white matter injury after ICH, possible via iron clearance [32, 36, 37, 64]. And ZnPP reduced white matter injury via reducing heme degradation products after ICH [29]. Our previous study indicated that the receptor for advanced glycation end-products (RAGE) antagonist alleviated axon injury in the white matter after ICH [11]. For further translational studies, more attention is needed to explore the repair mechanism and related drugs of the white matter injury after hypertensive ICH in the future.

As demonstrated above, progress has been made on the treatment of white matter injury after hypertensive ICH. On the other hand, the relevant research in this field is still scanty and the targeted treatment is also limited. The key to solve the problem is to explore in depth the white matter injury after hypertensive ICH.

## 4. Animal Model for White Matter Injury after Hypertensive ICH

Through reviewing the relevant studies, there are three main methods to construct the white matter injury model: microballoon infusion [19], collagenase injection [48], and autologous blood injection [13]. The advantage of the microballoon infusion model is that it could effectively simulate the mass effect of the hematoma. Thereby, it has contributed to the evaluation of the mass effect of hematoma on the white matter. The disadvantage is that it fails to address the potential effects of the blood components, metabolites, and subsequent substances released by the clot formation [65]. The advantage of collagenase injection model is that it could accurately simulate ICH in humans and avoid technical difficulties in handling blood [66]. Furthermore, it also simulates the hematoma expansion of continuous bleeding that occurs naturally in ICH patients [67, 68]. For this reason, it is more accurate to simulate the occurrence and development of white matter injury after ICH, while this model could produce excessive inflammatory response and other non-hemorrhage-related effects which could affect the assessment severity of white matter injury [25, 66]. The autologous blood injection could imitate the effects of an intracerebral hematoma in the brain while avoiding the disadvantage of the collagenase injection model, which is helpful for us to study the damage of the blood components and metabolites to white matter after ICH. Nevertheless, it does not accurately simulate the occurrence of hypertensive ICH as the cerebral vasculature was not disrupted [19].

Laboratory animals used for white matter injury studies after ICH include canines [69], pigs [11], cats [70], rabbits [71], rats [36], and mice [58]. Both rats and pigs were widely used in white matter injury research. Rats were the most widely used in the research of white matter injury thanks to their competitive prices, easy accessibility, and easy anesthetic operation. At the same time, the relatively smaller brain size makes them difficult to simulate well-developed white matter in human brain. Known for their large, gyrated brain and well-developed white matter, pigs are an ideal animal for the study of white matter injury after hypertensive ICH, for example, early perihematomal edema. And the study found that the large hematoma volume in pigs after ICH was more limited to the target area compared to other animal species [13, 72]. Pigs also have their challenges, especially relatively higher purchase price and a larger volume.

In summary, there is no ideal animal model to simulate the natural process of human white matter injury after hypertensive ICH at present. But each model could be used to study certain pathophysiological aspects of the white matter injury after hypertensive ICH. That is to say, on the one hand, in the future, we need to choose methods of structuring animal models according to different research purposes; on the other hand, continuous exploration and establishment are demanded as for the new animal models, which is more consistent with the natural process of human hypertensive ICH. And the new animal models should be easily induced, relatively cheap, convenient, and effective for the studies of the pathophysiological mechanisms of white matter injury and repair.

## 5. A New Research Paradigm of White Matter Injury after Hypertensive ICH 

### 5.1. Hypertension for the Susceptibility and Pathological Changes

It is known that hypertension is the most essential pathogenic factor of hypertensive ICH. Previous studies have found that chronic hypertension could induce hypertrophy of intracerebral arterioles by increasing the expression of the vascular ECM, like fibronectin, laminin, and collagen IV [73]. This is an indication that chronic hypertension could affect blood supply of the white matter before onset of ICH, which was confirmed by recent research. The study found that hypertension disrupts the structure and function of cerebral blood vessels, which leads to ischemic damage of white matter regions critical for cognitive function [74]. What is more, another cerebral finding in chronic hypertension was hypertensive encephalopathy, in which breakdown of the blood-brain barrier to serum proteins occurred in multifocal areas of the cortex and basal ganglia [75]. Mentioned earlier, the blood-brain barrier damage could lead to brain edema and then cause white matter injury. This prompted us to think about whether the treatment of chronic hypertension is helpful to reduce white matter injury after hypertensive ICH [76].

In addition to that, antihypertensive therapy after hypertensive ICH might also have a positive effect on relieving white matter injury. A study found that systolic blood pressure was substantially raised compared with usual premorbid levels after ICH [24]. However, previous researches indicated that lower blood pressure is not always good for ICH patients. In fact, after hypertensive ICH, the benefits of early treatment to reduce systolic blood pressure to 140 mmHg might be enhanced by smooth and sustained control and particularly by avoiding peaks in systolic blood pressure [77]. And as interpreted above, the severe hemodynamic change of the cerebral circulation could cause damage to the white matter after hypertensive ICH. Apparently, there exists a correlation between hypertension and white matter injury after hypertensive ICH.

To conclude, hypertension could induce white matter injury. Currently, research considered that ischemic damage was the main pathogenic mechanism of white matter injury caused by hypertension. However, the relationship between hypertension and white matter injury has not been further investigated after hypertensive ICH. The pathogenic mechanism of white matter injury caused by hypertension also needs in-depth exploration and discussion. Moreover, whether the treatment of chronic hypertension is useful to reduce the white matter damage after hypertensive ICH is an intriguing topic. It is enchanting that plenty of inspiring work is waiting to be discovered about hypertension and white matter injury.

### 5.2. Lenticulostriate Artery-Neural Complex

As indicated in previous sections, a number of pathogenic factors caused white matter injury after hypertensive ICH. Clearly, these pathogenic factors are interrelated. This reminds us that the white matter is simultaneously or sequentially damaged by different pathogenic factors after hypertensive ICH, not to mention that hypertensive ICH could also damage other brain tissues around white matter injury, like blood vessels and nerve nuclei. Evidently, these damaged brain tissues could conversely generate white matter injury. Far from it, through the review of the previous studies on white matter injury, it is the damage of the single pathogenic factor that these therapeutic studies focus on. Animal experiments mainly focus on the inflammatory response which is induced by the blood components and metabolites, while clinical trials are mainly emphasized in the mass effect of hematoma, and what these studies have in common is that they all did not pay attention to the effects of the damaged surrounding brain tissues on the white matter. Out of question, previous studies have improved our understanding of white matter injury after hypertensive ICH. And based on these studies, we have identified dozens of therapeutic agents and methods for the treatment of white matter injury after hypertensive ICH. And yet we should also take notice that many drugs and methods are rarely used in clinical practice. Therefore, we need to rethink our research strategy on white matter injury after hypertensive ICH.

**Figure 2:**
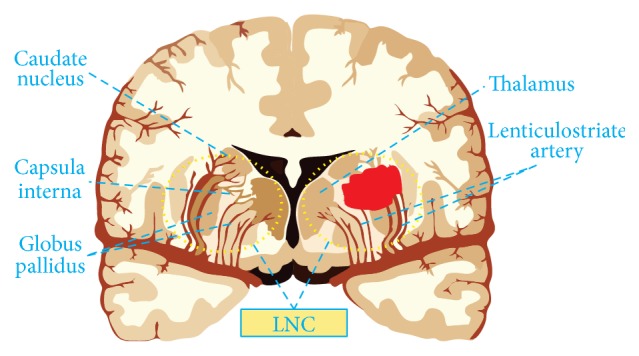
Illustrations for the lenticulostriate artery-neural complex.

In the past 20 years, there has been a dramatic change in the research paradigm of stroke pathophysiology. For example, preliminary studies found that neuroprotection alone for ischemic stroke could not yield a benefit. Thus, the neuronal-astrocytic-vascular tripartite functional unit was initially proposed in 1996 [78]. Cohen and others believed that dysfunctions in these neurovascular interactions might result in perfusion deficits and might be involved in specific pathological conditions [78]. This concept was revised and named the neurovascular unit at the first Stroke Progress Review Group meeting in 2001 [79]. The neurovascular unit emphasized the complexity of interactions between all perivascular cell types [80]; and it integrated neural and vascular cell types to help explain the failure of neuroprotective strategies for ischemic stroke [81]. But the neurovascular unit model focuses largely on the areas immediately surrounding capillaries, where neural and vascular cells interact and influence each other, and excludes downstream venous vasculature, upstream arterioles, and smaller arteries [80–82]. In 2012, Zhang and others proposed the vascular neural network as a new paradigm that combines the original concept of the neurovascular unit with emerging understanding of the key roles of arterial smooth muscle cells, endothelial cells, and perivascular nerves in cerebrovascular physiology and pathology [81]. This paradigm prominently promoted the study of stroke. In view of it, the research paradigm, which focuses on pathogenic factor alone, could not accurately reveal the pathophysiology of white matter injury after hypertensive ICH. In consequence, based on the anatomic structures of the most common bleeding site, we propose the research paradigm of the lenticulostriate artery-neural complex (LNC) (Figure 2), which is highly expected to contribute to the study of white matter injury after hypertensive ICH.

Lenticulostriate artery-neural complex is composed of the lenticulostriate artery and its hemodynamic system, nerve nuclei of the hypothalamus-thalamus-basal ganglia system, and the white matter fibers were located within the capsula interna. With this specific and representative structure as research paradigm, the LNC has the following superiorities. To start with, it is instrumental to avoid the limitations of previous studies on white matter injury after hypertensive ICH. And it will make us pay more attention to the early warning of white matter injury, the role of biomechanics in the white matter injury, the overall protection of white matter and surrounding nerve nuclei after hypertensive ICH, and so forth. Secondly, not only could it explain why the predilection site of hypertensive ICH is the lenticulostriate artery, but also it could be used to clarify the role of the hemodynamic parameters changes and the stress boundary conditions of perivascular area in the white matter injury caused by hypertension. What is more, it is beneficial to the coupling of hemodynamics, endothelial cells, vascular smooth muscle cells, peripheral nerve nuclei, and so on. Additionally, the occurrence and development of white matter injury and repair after hypertensive ICH were studied from the cellular and molecular levels. Last but not least, it could also be used to illustrate the pathological changes and clinical manifestations of patients with hypertensive ICH. Just as a patient with hemiplegia, hemianopia and hemidysesthesia result from the white matter fibers injury within the internal capsule after hypertensive ICH; the mass effect of hematoma, hypothalamus injury, and brain edema formation are substantial factors leading to lethal hernia after hypertensive ICH; and long-term brain atrophy and cognitive impairment after hypertensive ICH are related to the toxicity of the blood components, metabolites, and excessive inflammation. In brief, the LNC could systematically take into account the roles and mechanisms of hemodynamics, blood vessel injury and rupture, hematoma stress injury, white matter fibers, nerve nuclei (hypothalamus, thalamus, and basal ganglia), and blood component and its metabolites; immunologic and inflammatory response in the onset and development of hypertensive ICH is of vital significance in the exploration of white matter injury and repair.

Research around the LNC is wholesome to display the mechanism of the occurrence and development of white matter injury and repair after hypertensive ICH. More importantly, it will contribute to the establishment of an early warning system, early diagnosis, and early intervention and repair strategies. Specifically, it mainly contains the following four aspects. Initially, we need to seek out the genetic and environmental risk factors and their interactions in the pathogenesis of white matter injury induced by hypertension and to establish an early warning molecular system. And then, we need to study the mechanism of hemodynamic and vascular coupling injury in the pathogenesis of hypertensive ICH and the specific imaging early warning signs in a state of intense change in blood pressure. Furthermore, we should work over the effect and mechanism of the stress of hematoma on the structure and function of the LNC, followed by providing a reliable scientific basis for the clinical intervention of white matter injury and meanwhile promoting repair. At last, it is also needed to investigate the role of excessive inflammatory response, which was a result of the toxic effects of blood, its metabolites, and the activation of the DAMPs, in the pathogenesis of the LNC lesion. Only in this way could we better understand the white matter injury after hypertensive ICH and its early warning and treatment.

On the whole, relatively fewer studies focused on white matter injury and repair after hypertensive ICH, and many issues still exist in the current research strategy. As a new research paradigm, the LNC helps us to better comprehend the pathophysiology of white matter injury and repair after hypertensive ICH.

## Figures and Tables

**Table 1:** The detection methods of white matter injury.

Detection method	Advantage	Disadvantage	Detection mode
Diffusion tensor magnetic resonance imaging	(1) Assessing longitudinal change in white matter fibers (2) Selectively visualizing the white matter fibers (3) Early detection of the white matter injury	(1) Expensive (2) Requires higher hardware and software	Direct

T1- and T2-weighted spin-echo sequences magnetic resonance imaging	(1) Wider application (2) Roughly assessing the damage situation of white matter	(1) Expensive (2) Unable to early detect the white matter injury (3) Unable to clearly visualize the white matter fibers	Direct

Computed tomographic scan	(1) Inexpensive (2) Wider application (3) Roughly assessing the damage situation of white matter	(1) Unable to early detect the white matter injury (2) Unable to clearly visualize the white matter fibers	Direct

Laser Doppler	(1) Inexpensive (2) Quickly and accurately detecting the blood flow changes of white matter	(1) Unable to directly detect the white matter injury (2) Invasive examination	Indirect

Pathological examination	(1) Cheap (2) Has a variety of detection means (3) Quickly and accurately finding the change in white matter fibers	Invasive examination	Direct

Radioactive microsphere technique	Quickly and accurately detecting the blood flow changes of white matter	More difficult to apply in clinic	Indirect

Poststroke activities of daily living (ADL)	(1) Easy to use and cheap (2) Quickly detecting white matter injury	Only indirectly and roughly judged white matter injury	Indirect

Informant Questionnaire on Cognitive Decline in the Elderly (IQCODE)	(1) Easy to use and cheap (2) Quickly detecting white matter injury	Only indirectly and roughly judged white matter injury	Indirect

Modified Telephone Interview for Cognitive Status test	(1) Easy to use and cheap (2) Quickly detecting white matter injury	Only indirectly and roughly judged white matter injury	Indirect
